# Second Toe Transfer and “Tandem” Free Flap for Radial Hand Injuries with Thumb Loss

**DOI:** 10.1055/s-0045-1810048

**Published:** 2025-08-13

**Authors:** R. Srikanth, D. Mukunda Reddy, R. Parvathi, N. Rambabu, Muralimohana Reddy K., V. Bhanuchander, Babu Rao R

**Affiliations:** 1 Department of Plastic and Reconstructive Surgery, Nizams Institute of Medical Sciences, Hyderabad, Telangana, India; 2 Department of Plastic and Reconstructive Surgery, Basavatarakam Smile Train Centre, Hyderabad, Telangana, India; 3 Department of Plastic Surgery, Basavatarakam Cancer Hospital, Hyderabad, Telangana, India; 4 Department of Plastic Surgery, Yashoda Hospitals, Secunderabad, Telangana, India; 5 Department of Plastic Surgery, Elite Hospitals, Tirupati, Andhra Pradesh, India

**Keywords:** second toe transfer, free flap, thumb amputation, thumb reconstruction

## Abstract

**Materials & Methods:**

Eleven cases of thumb loss and skin defect in the radial aspect of the hand underwent a simultaneous free flap cover and second toe transfer. Seven were emergency and four were elective. Nine of the flaps were the anterolateral thigh (ALT) flap and two were extended versions of the lateral arm flap. The mean area of tissue defect needing replacement with the free flap was 110 cm2. All the toe transfers used the second toe. The flap revascularization preceded that of the toe.

**Results:**

The descending branch artery of the ALT flap or a branch of the radial artery, if present, was used to revascularize the transferred toe. The venous anastomosis to the flap and the toe used a combination of the superficial veins and venae comitantes of the radial pedicle. Though there were two re-explorations, only one transferred toe was lost.

**Discussion:**

Among the ten patients who were evaluable at follow up, the opposition as measured by the mean Kapandji score was 6.

**Conclusion:**

All patients had useful grasp and thumb pinch. Secondary procedures like flap thinning and tenolysis were done in seven cases.

## Introduction

There are various methods to reconstruct a lost thumb, in part or whole, when replantation is not feasible or unsuccessful. Amputations close to the interphalangeal joint (IPJ) can undergo distraction lengthening to increase thumb length to a functional level; this may need provision of good quality skin preferably sensate. This method does not provide for a nail–pulp complex. 1

For more proximal thumb amputations, skeletal lengthening of the remnant using bone grafts and skin flap coverage has been the “osteoplastic” method to reconstruct the lost thumb. Traditionally, the flap coverage preceded the bone grafting, but authors have shown successful results with flap cover and one-stage immediate bone grafting also. 2

The emergency transfer of toes to reconstruct a thumb amputation has been proved to be both safe and effective and enables an earlier return of function. 3

Variants of the “toe transfer” are a sophisticated “like for like” reconstruction method and a preliminary flap cover in the presence of significant tissue loss ensures adequate availability of skin at the time of the definitive procedure. The use of a pedicle flap, usually the groin flap, implies a two-stage procedure with a delay in achieving hand function and patient rehabilitation. 4

In addition to the loss of the thumb, there may be a loss of other radial digits with or without fractures that severely compromise hand function. If the defect is large for the use of a pedicle flap, then using a free flap and doing the toe transfer simultaneously could help in optimal use of the recipient vessels. 5

Though the great toe transfer gives a better appearing neo-thumb, it comes at a significant donor cost. The second toe transfer can provide reasonable neo-thumb function with a much lesser impact at the donor site.

Double free flaps have been used in reconstruction of other defects, especially in the head and neck and have been considered safe. 6 The use of flow through flaps like the anterolateral thigh (ALT) has made possible sequential microvascular anastomosis of multiple flaps using a single recipient vessel. 7

This technique of combining tissue addition and thumb reconstruction in the same stage could ensure the earliest return to a rehabilitated equilibrium.

## Aims and Objectives

This was twofold: (1) the feasibility of successful transfer of the second toe and a free flap simultaneously to the radial side of the hand; (2) the evaluation of function and appearance at follow-up.

## Materials and Methods

**Fig. 1:**
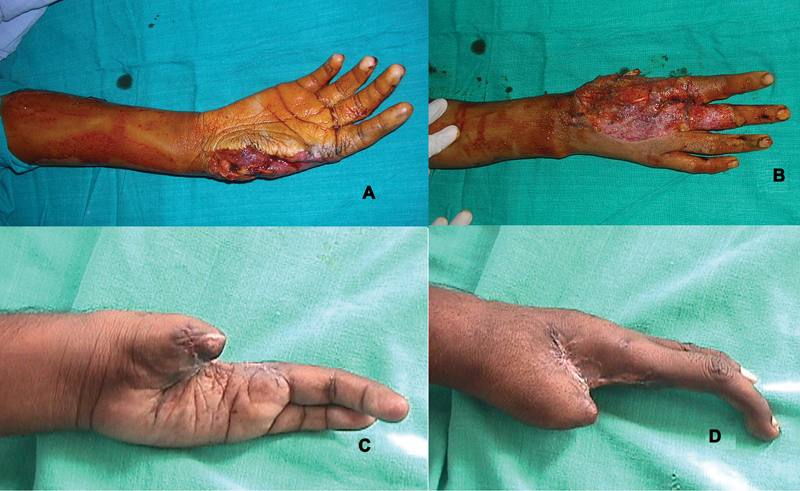
( **A, B** ) Dorsal and volar views of thumb loss at proximal metacarpal with skin loss of radial aspect of the hand—emergency presentation; ( **C, D** ) dorsal and volar views of thumb loss at distal metacarpal with scarred first web- elective presentation.

This is a consecutive series of 11 cases of trauma with skin loss in the radial aspect of the hand and loss of thumb, of which seven were emergency and four were elective reconstructions ( Fig. 1 ).

All patients had a simultaneous free-flap cover and thumb reconstruction using the second toe transfer. There were 9 males and 2 females with age ranging from 24 years to 42 years. Six patients had workplace injury by industrial machinery and five were the sequelae of road traffic accidents ( Table 1 ).

**Table 1:** Injury details

	Type	Mode of injury	Level of thumb loss	Loss of skin	Other injuries
Case 1	Emergency	Road traffic accident	Proximal phalanx base	14 × 8 cm Dorsum radial hand and first web	Metacarpal fractures: 2, 3, 4
Case 2	Emergency	Industrial machinery	Proximal phalanx base	8 × 8 cm first web	Amputation index and middle finger at metacarpal neck
Case 3	Elective	Road traffic accident	Through middle of metacarpal	Scarred grafted stump absent first web; 10 × 8 cm	Skin graft over stump and first web; index metacarpal loss
Case 4	Emergency	Industrial machinery	Through middle of metacarpal	Loss of first web and contiguous dorsum 12 × 8 cm	Failed partial hand replantation; amputation index middle and ring finger
Case 5	Emergency	Industrial machinery	Through proximal metacarpal	18 × 10 cm First web, volar wrist, palm and distal forearm	Degloving of distal third forearm and loss of palmar skin; metacarpal fractures
Case 6	Elective	Road traffic accident	Through proximal metacarpal	Loss of first web and contiguous dorsum hand 12 × 8 cm	Index finger amputation
Case 7	Elective	Industrial machinery	Through distal metacarpal	Loss of first web 10 × 8 cm	Index and middle finger amputation
Case 8	Emergency	Industrial machinery	Through proximal metacarpal	14 × 10 cm Volar and dorsal wrist	Multiple volar wrist laceration with degloved dorsal hand
Case 9	Elective	Road traffic accident	Through middle of metacarpal	10 × 8 cm Loss of first web and scarred radial dorsum hand	Insensate and immobile index with flexion contracture PIPJ middle finger
Case 10	Emergency	Road traffic accident	Through middle of metacarpal	Loss of first web and degloving dorsum 12 × 10 cm	Second metacarpal fracture
Case 11	Emergency	Industrial machinery	Through distal metacarpal	Loss of first web and degloving dorsum 12 × 10 cm	Second metacarpal fracture

Abbreviation: PIPJ, proximal interphalangeal joint.

**Fig. 2:**
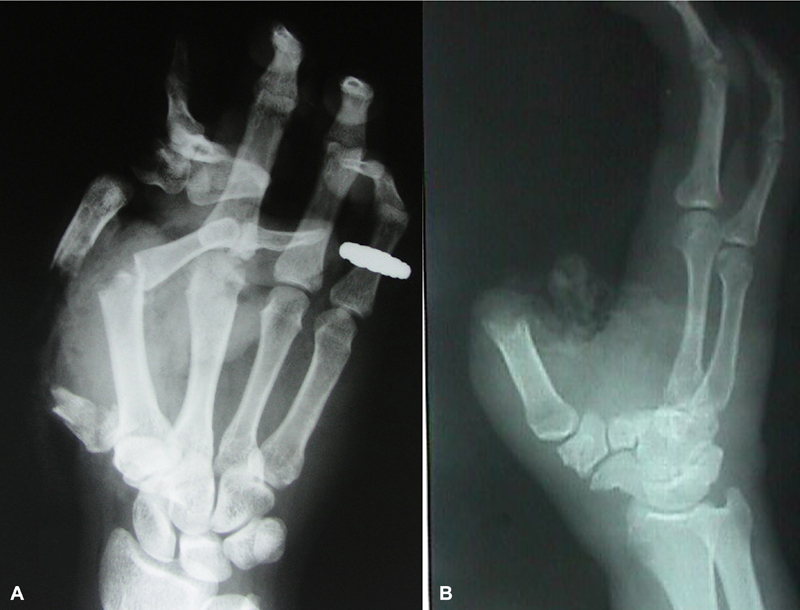
Preoperative X-rays of the first and second patient in Fig. 1 ( **A** and **B** ).

None of them had injuries other than the involved hand, and after all relevant investigations to judge fitness for anesthesia, were graded as American Society of Anesthesia Grade I. Patients with injuries of other limbs, polytrauma victims, and severely degloved hands were excluded from this single-stage reconstruction. Relevant X-rays were done to define the level of skeletal loss of the thumb ( Fig. 2 ).

In all of seven emergency injuries, the necessary debridement was done under brachial block and thence posted for semi-elective definitive procedure within the next 24 hours. Two of the seven emergency cases were following a failed attempt at replantation or revascularization. Associated injuries in the same hand included crush amputations of neighboring digits and metacarpal or phalangeal fractures.

The patients were explained about the advantages and disadvantages of a single-stage procedure to replace the lost thumb using the second toe and the need for a free flap for coverage; the advantage of primary approximation of the foot after a second toe transfer, resulting in better aesthetic appearance of the foot and the ability to continue usage of Indian-style conventional footwear, was stressed upon. The possibility of re-exploration and loss of transferred tissues was also explained. The fact that the replaced toe would give the advantage of span grasp, power grip, and thumb–index pinch in a single operation was conveyed to the patient. The need for secondary procedures to improve function and/or appearance after a suitable follow-up period was also told.

All the toe transfers used the second toe; 2 of the 11 were a second toe wraparound type of transfer and other 9 were whole second toes. Two of the 11 flaps were the extended lateral arm flap and 9 were ALT flaps ( Table 2 ).

**Table 2:** Operative details

	Flap	Toe	Recipient artery for flap	Recipient artery for toe	Recipient veins	Donor closure
Case 1	ALT	Second toe excluding MTPJ	Radial artery	Descending branch ALT	Flap: tributary cephalic Toe: cephalic and radial venae comitantes	Flap donor site: split skin graft Toe donor site: primary closure
Case 2	Extended lateral arm	Second toe wraparound	Superficial branch radial artery	Deep branch radial artery	Flap: radial venae comitantes Toe: cephalic and radial venae comitantes	Flap donor site: primary closure Toe donor site: primary closure
Case 3	ALT	Second toe including MTPJ	Radial artery	Descending branch ALT	Flap: tributary cephalic Toe: cephalic and radial venae comitantes	Flap donor site: split skin graft Toe donor site: primary closure
Case 4	ALT	Second toe including MTPJ	Radial artery	Descending branch ALT	Flap: tributary cephalic Toe: cephalic and radial venae comitantes	Flap donor site: split skin graft Toe donor site: primary closure
Case 5	ALT	Second toe including MTPJ	Radial artery	Descending branch ALT	Flap: tributary cephalic Toe: cephalic and radial venae comitantes	Flap donor site: split skin graft Toe donor site: primary closure
Case 6	ALT	Second toe including MTPJ	Radial artery	Descending branch ALT	Flap: tributary cephalic Toe: cephalic and radial venae comitantes	Flap donor site: split skin graft Toe donor site: primary closure
Case 7	Extended lateral arm	Second toe excluding MTPJ	Radial artery terminal branch	Radial artery terminal branch	Radial artery terminal branch	Flap donor site: primary closure Toe donor site: primary closure
Case 8	ALT	Second toe including MTPJ	Radial artery	Descending branch ALT	Flap: tributary cephalic Toe: cephalic and radial venae comitantes	Flap donor site: primary closure Toe donor site: primary closure
Case 9	ALT	Second toe including MTPJ	Radial artery	Descending branch ALT	Flap: tributary cephalic Toe: cephalic and radial venae comitantes	Flap donor site: primary closure Toe donor site: primary closure
Case 10	ALT	Second toe excluding MTPJ	Radial artery	Descending branch ALT	Flap: tributary cephalic Toe: cephalic and radial venae comitantes	Flap donor site: split skin graft Toe donor site: primary closure
Case 11	ALT	Second toe wraparound	Radial artery	Descending branch ALT	Flap: tributary cephalic Toe: cephalic and radial venae comitantes	Flap donor site: split skin graft Toe donor site: primary closure

Abbreviations: ALT, anterolateral thigh; MTPJ, metatarsophalangeal joint.

## Surgical Technique

### Emergency Reconstruction

**Fig. 3:**
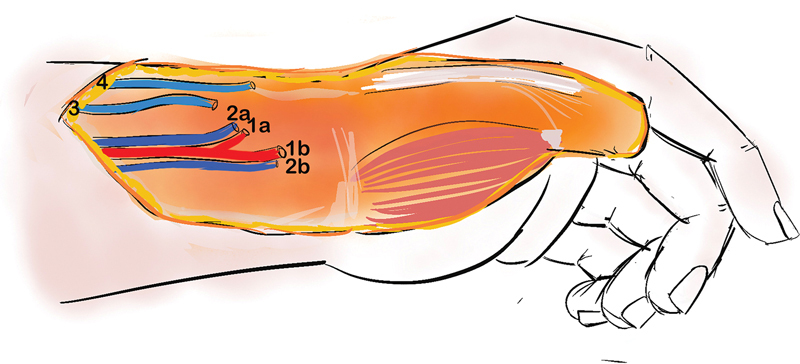
Recipient pedicle dissection plan. 1a, 1b: two branches of radial artery; 2a, 2b: two venae comitantes of radial artery; 3, 4: two superficial veins in the forearm.

**Fig. 4:**
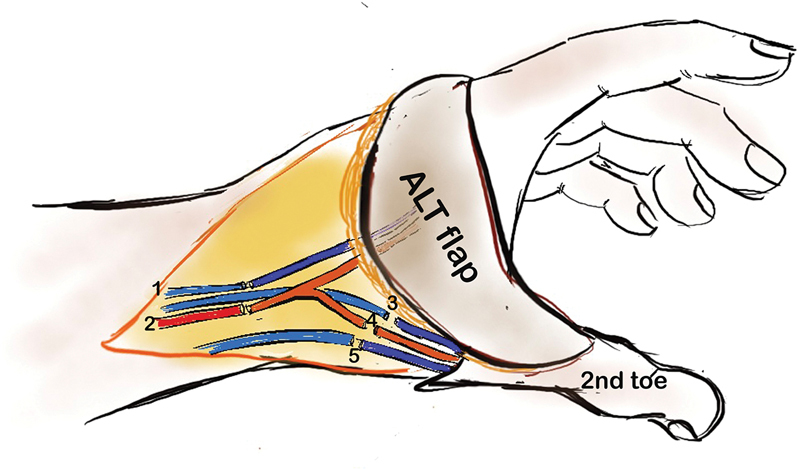
Alternative anastomotic plan. 1: superficial vein to ALT flap vein; 2: radial artery to ALT flap artery; 3: venae comitantes to dorsalis pedis venae comitantes; 4: descending branch artery to dorsalis pedis; 5: superficial vein forearm to great saphenous vein. ALT, anterolateral thigh.

The debridement was done in a preliminary operation under brachial block to define the degree of injury, without any attempt at dissecting the recipient pedicle. The definitive surgery started with recipient pedicle dissection under tourniquet control, proximal to the zone of trauma, identifying the radial artery, its branches, the cephalic, and other superficial veins nearby. The choice of the recipient artery depended on the proximal extent of skin loss; where the injury was restricted to the hand, both the radial artery branches would be available and where the injury extended more proximally, the descending branch of the lateral circumflex femoral served as the arterial inflow ( Figs. 3 and 4 ).

The extrinsic thumb flexors and extensors were tagged proximally, as were the volar digital nerves issuing from the median nerve. If the extrinsic tendons of an amputated index finger were healthier than that of the native thumb, they were preferred as the donors. The proximal remnant of the thumb skeleton was prepared (at the proximal phalanx base or the metacarpal as per the injury level) for fixation. The proposed length of the thumb was taken from the opposite side. A template of the skin defect was taken to be transferred to the donor site (either the opposite thigh or the lower lateral arm). The ipsilateral second toe (as the flap donor site was opposite to the injured hand) was chosen to facilitate the simultaneous harvest of toe and flap, and this started after preparation of recipient site was complete.

While elevating the ALT flap, at least an inch of the distal continuation of the descending branch of the lateral circumflex femoral vessels was harvested to permit it to be used as arterial inflow for the second toe. All the lateral arm flap harvests were of the extended lateral arm flap configuration, to gain pedicle length and utilize the thinner skin below the elbow. The lateral arm flap elevation was done with a 4-inch Esmarch bandage at the root of the arm.

The second toe transfer was done under exsanguination and tourniquet control, after a preliminary palpation and Doppler to estimate the type and location of the first dorsal metatarsal artery. The toe dissection started proximally from the dorsum, following the vessels before the plantar dissection to isolate the plantar digital nerves and the flexor tendon. Skeletal section of the toe depended on the level of the thumb loss. Where the base of the proximal phalanx was present, the toe was transferred through its proximal phalanx and where the loss was at the metacarpal, the toe metatarsal was included. Once the toe was isolated on its vascular pedicle, strategic placement of micro-clamps confirmed which of the dorsal or plantar systems was dominant. This was done after tourniquet deflation and facilitated critical reperfusion of the toe before detachment. Among all the toe transfers, only one case had a plantar-dominant arterial system needing an anastomosis of the plantar digital artery to the recipient artery using a short vein graft.

**Fig. 5:**
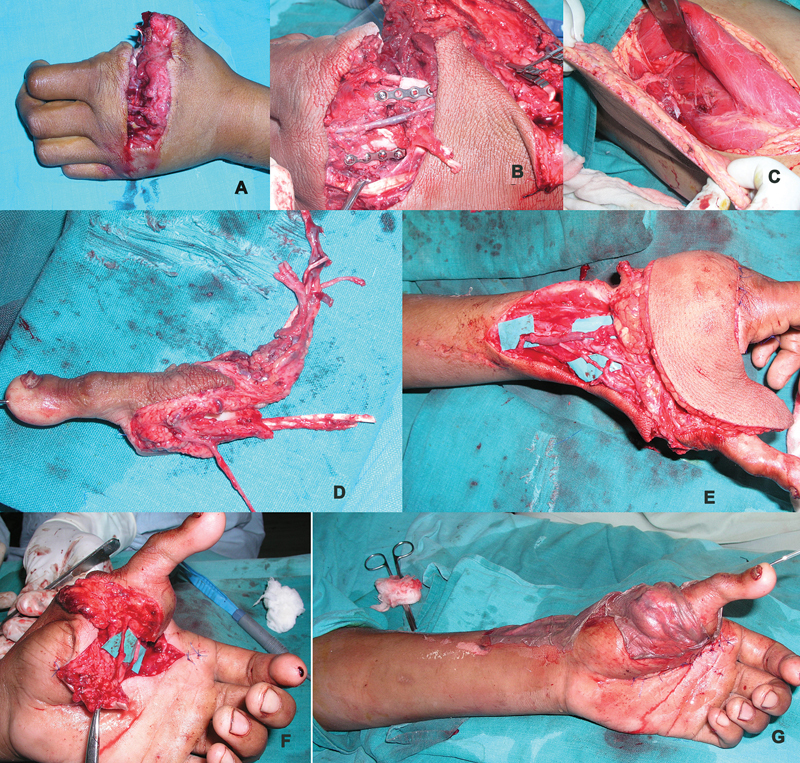
( **A, B** ) Dorsal skin loss, amputated thumb, and multiple metacarpal fractures fixed after debridement with miniplates; ( **D** ) harvested ALT flap before detachment; ( **D** ) second toe after detachment; ( **E** ) at completion of vascular anastomosis and before complete flap inset; ( **F** ) nerve repair of volar digital nerves of the transferred toe; ( **G** ) split skin graft over recipient pedicle and residual raw areas. ALT, anterolateral thigh.

**Fig. 6:**
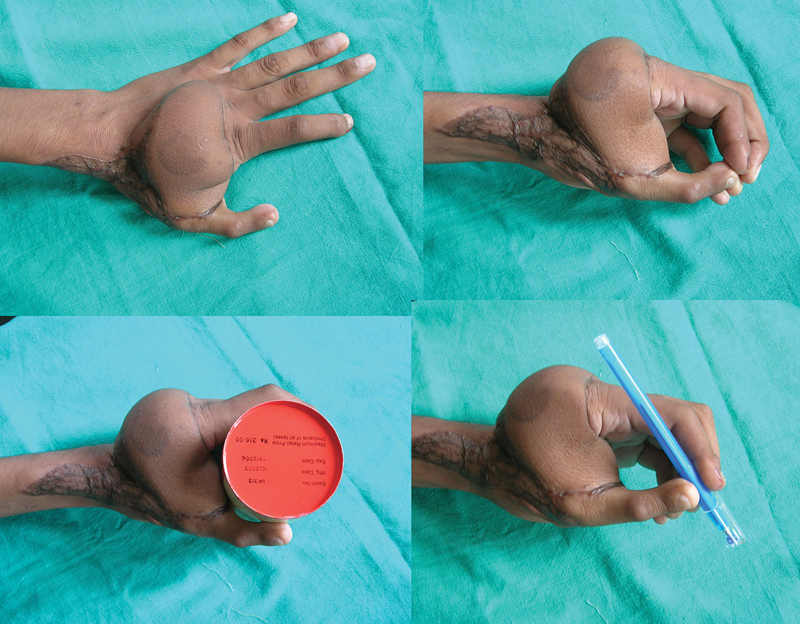
Images at follow-up of the patient in Fig. 8 , before flap thinning showing span grasp and precision grip between thumb and index fingers.

The flap was transferred first to the hand and anastomosis completed, as in all the ALT flaps, the descending branch artery served as the inflow for the toe. When the lateral arm flap was used, then the branches of the radial artery were available to independently perfuse the flap and the toe; still it was the flap that was transferred first. A single venous anastomosis to the available superficial veins was done for all the transferred skin flaps ( Figs. 5 and 6 ).

**Fig. 7:**
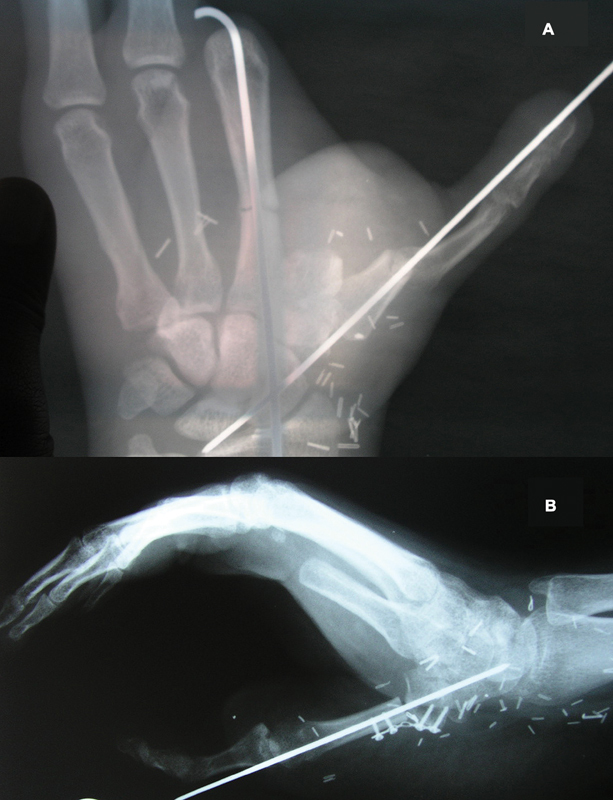
( **A** ) Postoperative X-ray with fixation using longitudinal K wire; ( **B** ) postoperative X-ray with fixation using longitudinal K wire and miniplate fixation.

Once flap perfusion was confirmed, the toe was detached and transferred to the hand, after the retrograde passage of a longitudinal K wire, that could be driven antegrade onto the thumb skeletal remnant. Where the level of thumb amputation was at the proximal metacarpal level, addition of a titanium miniplate afforded better stability, as most of the thumb intrinsics would have been lost ( Fig. 7 ).

A repair of the extrinsic flexors in standard tendon repair technique preceded the vascular anastomosis—this was to the dorsal artery in all cases except one. The venous anastomosis was always two—the saphenous vein to the superficial vein and the venae comitantes of the dorsalis pedis to the radial venae comitantes. The descending branch veins were never used for the venous anastomosis of the toe to keep them independent. The extrinsic extensor tendon repair was done after the microvascular anastomosis. Nerve coaptation preceded wound closure.

### Note

- In the only case where the plantar arterial system of the toe was dominant, the plantar digital vessels were anastomosed to the radial artery using a 7 cm interposition venous graft harvested from the opposite forearm.
- Unexplained venous congestion of the toe during surgery was seen in one case, and it was presumed to be due to poor quality of superficial vein at the site of anastomosis; this was expedited by taking down the anastomosis and interposing a 15 cm saphenous vein graft from the opposite leg and connecting the superficial vein of the toe to healthier superficial veins at the cubital fossa; this succeeded in relieving the venous congestion.

If the area over the recipient pedicle could not be closed for fear of compression, a split skin graft was used.

The toe donor site was always closed primarily, over a suction drain, paring the second metacarpal remnant to its base. A below-knee plaster of Paris slab with foot in plantigrade position was added. If the flap donor site could not be closed primarily, it was split skin grafted. A plaster of Paris slab was preferred to immobilize the elbow after an extended lateral arm flap harvest.

#### Elective reconstruction

**Fig. 8:**
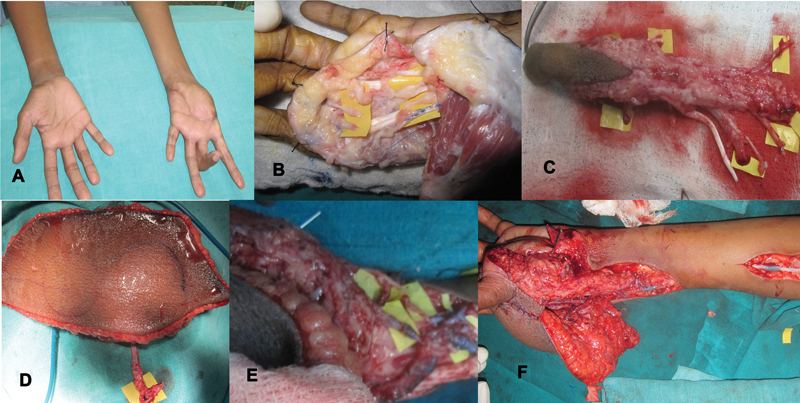
( **A** ) Loss of thumb and first web space with flexion contracture middle finger following previous trauma; ( **B** ) recreation of defect after scar excision and deformity correction for index and middle fingers; ( **C, D** ) second toe and ALT flap harvested; ( **E, F** ) at completion of venous anastomosis, unexplained venous congestion of toe necessitated use of a vein graft to bridge toe veins to cubital fossa vein, successfully relieving the venous congestion. ALT, anterolateral thigh.

**Fig. 9:**
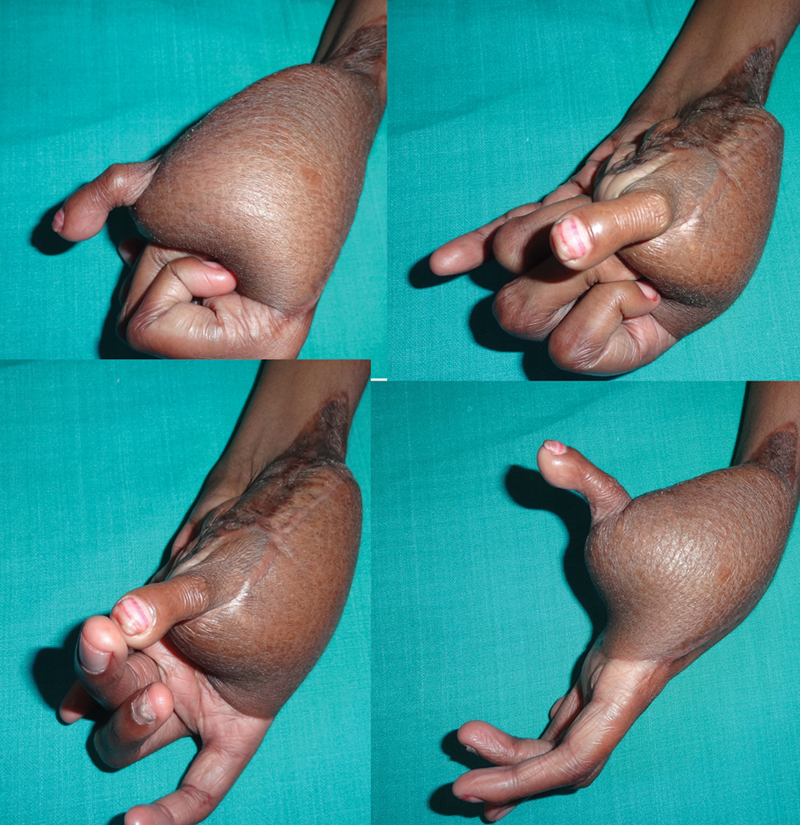
Follow-up images of the patient in Fig. 12 , prior to flap thinning showing span of first web and Kapandji score of 5; limited active movement range of interphalangeal joint noted.

Except that the skin defect had to be created to receive the flap, all the other steps were identical to the emergency reconstructions ( Figs. 8 and 9 ).

All the patients received 12-hourly infusion of low-molecular-weight dextran in saline solution in the postoperative period for 3 days. The use of this was based only on empiric evidence. No other form of anticoagulation was used, except heparin saline solution for irrigation during the vascular anastomosis.

The patients were discharged between the 10th and 15th postoperative day, in a below elbow plaster of Paris slab, and this was removed at 3 weeks. The K-wire was removed between 6 and 8 weeks after X-ray imaging. Active and passive therapy started after this period. Scar massage and purposive activities to simulate daily living activities were advised after 12 weeks, with instructions not to touch any hot object for another 6 months.

## Results

### Immediate and Early Postoperative Period

- Redo anastomosis: there was one redo arterial anastomosis due to anastomotic block in the toe vessel. One other case (described in surgical technique) needed redo venous anastomosis with an interposition vein graft for the superficial vein of the toe.
- Re-exploration: one case needed revision anastomosis of the toe using a vein graft; though successful on table the venous congestion returned and the toe could not be salvaged. The flap in this case survived but the thumb reconstruction failed. The only other re-exploration was on account of venous congestion of the flap and was successfully tackled by draining a hematoma at the site of the recipient pedicle and suitable hemostasis.
- Four of the flap donor sites could be closed primarily and healed well. One of the 7 split skin-grafted donor sites (ALT donor) had graft loss managed expectantly with delayed healing.
- All except two of the toe donor sites healed completely by 10 days; the healing period in these two cases was prolonged to 5 weeks but no secondary procedure was needed.
- Ten of the 11 reconstructions were successful at the time of discharge ( Table 3 ).

**Table 3:** Outcome details

	Immediate outcome	Follow-up duration	TAM in degrees (except basal joint)	Kapandji score	Secondary procedures
Case 1	Complete survival	20 months	50	8	1 flap thinning
Case 2	Complete survival	36 months	40	8	1 flap thinning
Case 3	Complete survival	22 months	40	5	Extensor tenolysis
Case 4	Complete survival	20 months	30	6	–
Case 5	Re-exploration for flap venous congestion due to hematoma; drainage and hemostasis relieved the venous congestion; complete survival	9 months	20	4	–
Case 6	Complete survival	14 months	30	7	Flap thinning, flexor tenolysis
Case 7	Complete survival	20 months	30	6	–
Case 8	Re-exploration for venous congestion toe, use of vein graft, re-block and toe could not be salvaged but flap survived	–	–	–	No further reconstruction after loss of toe
Case 9	Intraoperative venous congestion toe, use of vein graft from toe vein to superficial vein at cubital fossa bypassing injured segment, complete survival	18 months	30	5	1 flap thinning, MPJ manipulation and splintage
Case 10	Complete survival	19 months	40	6	Flexor tenolysis
Case 11	Intraoperative arterial block of flap artery, revised on table with complete survival	28 months	30	6	1 flap thinning

Abbreviations: MPJ, metacarpophalangeal joint; TAM, total active motion.

### Follow-Up

The follow-up on the 10 successful reconstruction ranged from 9 months to 7 years; 2 of the 10 patients did not follow up beyond 9 months. Secondary surgery was done in seven cases. There were 5 flap-thinning procedures, 2 extrinsic flexor tenolysis, 1 extrinsic extensor tenolysis, and 1 joint manipulation and splintage under anesthesia.

The authors evaluated total active motion (TAM; for the IPJ and the metacarpophalangeal joint [MPJ]) and Kapandji score to assess functional utility of the neo-thumb. This was considered by assessing the response to fine touch using cotton wool and when final functional outcome was being assessed, the response of patient to recognize everyday objects with eyes closed. Only one patient had poor return of sensation; redo surgery for improving sensation was advised but the patient refused. The Kapandji score ranged from 4 to 8 with a mean value of 6.

## Discussion



**Video 1** Follow-up function after emergency second toe wraparound and extended lateral arm flap.


**Video 2** Follow-up function after elective second toe transfer and ALT flap. ALT, anterolateral thigh.


**Video 3** Follow-up function after elective second toe transfer and ALT flap. ALT, anterolateral thigh.


Woo et al 8 described a series of 25 immediate toe transfers for thumb and digital amputations where either replantation was not feasible or was unsuccessful. Though the majority of these were either a great toe transfer or great toe wrap around flap, all the transfers were successful. There was no difference in re-exploration rates or infection. Forty four percent of the immediate reconstruction group were able to retain their original jobs as against 26% in the delayed thumb reconstruction group. The authors caution about meticulous debridement and doing the toe transfer in a semi-elective manner after 24 to 48 hours of the primary debridement, to prevent complication of infection and toe loss. Since none of the thumb losses were proximal to the Metacarpophalangeal (MCP) joint and the first web skin was uninvolved, the issue of flap coverage did not come up.

Ornelli et al 9 also found no difference in toe loss or recipient-site infection in two groups of patients with immediate as against delayed toe transfer. Avulsion injuries of the radial side of the hand resulting in thumb amputation were excluded in this series.

In the present series, where the procedure was for an emergency indication, we allowed at least 12 hours to elapse between the debridement and the definitive reconstruction; this permitted us to remove any questionable areas of skin loss prior to the flap and toe transfer.

The distally based radial forearm flap has been elegantly described both as a source of skin replacement and vascular inflow for a toe transfer by Kim et al 10 ; three were emergency and five were elective. Though the authors do not provide details of the skin defect, it can be assumed that limited skin defect in the area of the first web could be covered with this flap. The reverse nature of the flap implies retrograde venous drainage through the venae comitantes and the need for an antegrade cephalic vein in addition by excluding the cephalic vein from the flap harvest.

Injuries can result in larger area of skin loss with complete loss of the first web and the level of thumb amputation may be proximal to the MCP joint. The toe can be harvested with extra skin from the dorsum of the foot for its use in the hand but usually this is at a cost to the foot defect which cannot be closed primarily.

del Piñal et al 11 report four emergency toe transfers for thumb reconstruction where the issue of skin coverage was solved by harvesting the fascio-subcutaneous tissue from the dorsum of the foot in continuity to the toe to avoid using another free flap. The limitation of size below 5 × 5 cm is stressed to avoid increasing morbidity at the donor site.

Zhang et al 12 published six cases of partial or total thumb reconstruction albeit using the great toe variants in tandem with free dorsalis pedis and anterior tibial skin flap. These defects of loss of thumb and scarred first web were the sequelae of trauma or burns. The flap donor sites need split skin graft over the dorsum of the foot and the lower anterior leg. This single-stage chimeric flap transfer yielded a functional thumb reconstruction with a change in 4.8 in thumb opposition Kapandji scores.

**Fig. 10:**
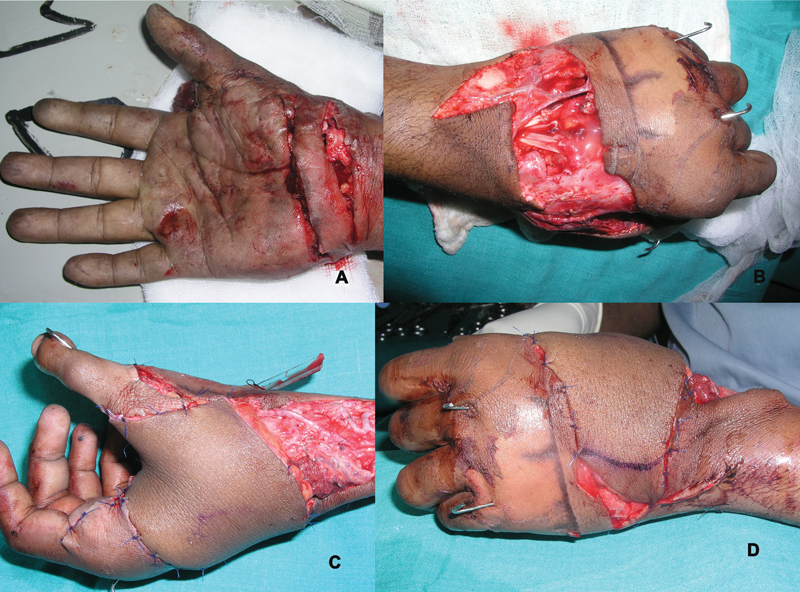
( **A** , **B** ) Devascularized multi-segment thumb injury with volar lacerations and loss of first web skin; ( **C** , **D** ) images after transfer of second toe and ALT flap for the dorsum and the first web. ALT, anterolateral thigh.

**Fig. 11:**
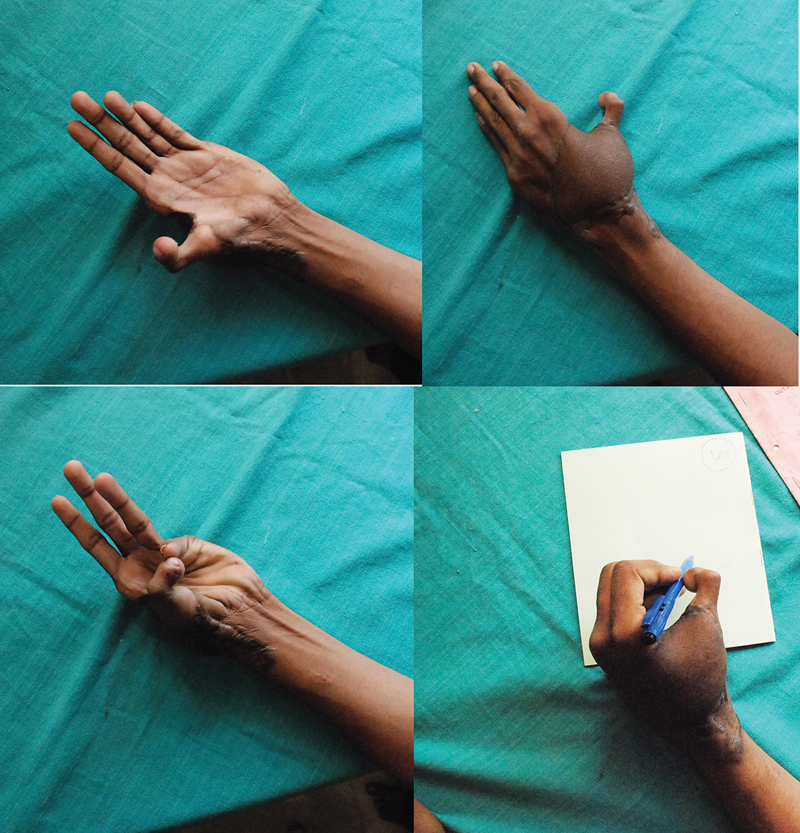
Follow-up images of the patient in Fig. 8 showing useful thumb opposition and functional precision grip; he underwent secondary surgery for flexor tenolysis to correct interphalangeal joint flexion deformity.

In the present series, the average size of the skin defect was 110 cm ^2^ and concerns about donor-site morbidity restricted the choice of flap to an area beyond the foot. The larger defects needed the ALT flap and the smaller, the extended lateral arm flap. Further, the complexity inherent in the design and inset of a chimeric flap as described by Zhang et al can be avoided by choosing a flap not based on the anterior tibial–dorsalis pedis system. The vascularity of the donor bed where the dorsalis pedis skin has been harvested is likely to be tenuous leading to a sub-optimal split skin graft take, with its implications for poor wound healing and donor morbidity. Choosing a different flap donor site, as we have done, limits the foot morbidity and places no restriction on the amount of skin harvested ( Figs. 10 and 11 ).

Rui et al 5 reported the first series of a combined free flap and toe transfer in 14 cases—12 were emergency and 2 were elective. The choice of toe was the second toe in all proximal amputations of the thumb, 10 in number, and the ALT or the lateral arm flap for cover depending on tissue requirements. The size of the flaps ranged from 30 to 350 cm ^2^ . Typically, the anastomosis to the toe was done first and the deep plantar branch in the foot served as the inflow for the pedicle of the flap. There was loss of only one toe. On long-term follow-up, there was satisfactory grasp, pinch, and opposition with a two-point discrimination (2 PD) of 4 to 8 mm. The authors also caution about poor return in opposition when the level of the thumb amputation is very proximal (6th grade of the Merle classification). 13

Since both the ALT and the lateral arm flaps are type III fasciocutaneous flaps, the distal continuation of the axial vessel can serve as the inflow for the toe transfer. We prefer to the revascularize the ALT flap first and use the descending branch artery for perfusing the toe; the descending branch is likely to be longer and with a bigger diameter than the deep plantar branch of the dorsalis pedis. During recipient vessel dissection, an effort is made to look for the presence of the superficial branch of the radial artery—this would match the vessels of the lateral arm flap if it were chosen. The venous anastomosis is always kept independent using a combination of the radial venae comitantes, the cephalic vein, and other superficial veins.

Fan et al 14 recommend the use of the deep plantar branch or the plantar arterial arch for flap perfusion with the radial artery reserved for the toe transfer. This series included 11 elective reconstructions using the ALT of the scapular flap and the first or the second toe. They report a great functional utility of the procedure using the Michigan Hand outcome scores and a change in first web from 0 before operation to 4.3 cm at follow-up.

With an average Kapandji score of 6 in the present series, most of the patients were capable of using the neo-thumb for grasp and pinch. This can be seen in the images and videos, as a formal hand function assessment using the Michigan Hand score was not done. The mean TAM was only 35 degrees—the reason could be the extension nature of the toe MPJ, the loss of intrinsic musculature in cases with amputation at proximal metacarpal levels leading to poor MPJ flexion, and propensity for flexor pollicis longus or extensor pollicis longus adhesions ( Videos 1 2 3 ).

Re-exploration rate and loss of toe (in one case) in the present series are comparable to reports by Rui et al. 5 Far more significant is the fact that in one elective toe and flap reconstruction, the venous congestion noted in the toe after completion of the anastomosis compelled us to use a vein graft to bridge the toe vein to normal superficial veins near the cubital fossa resulting in 100% survival of the transferred toe. This proactive stance during the surgery can avoid a less than optimal result after a postoperative re-exploration.

**Fig. 12:**
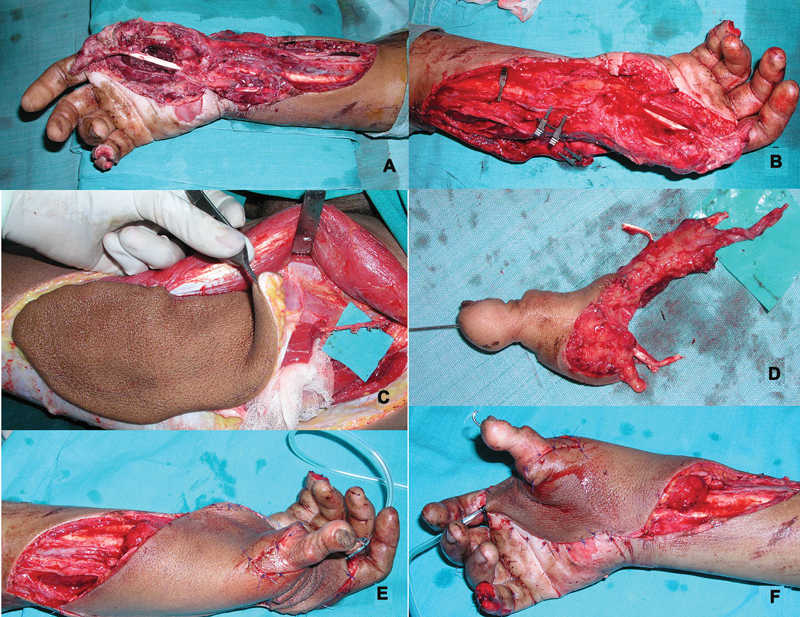
( **A** , **B** ) Extensive avulsion with loss of thumb near CMC joint; ( **C** , **D** ) harvested second toe and ALT flap; ( **E** , **F** ) at completion of flap and toe inset after microvascular anastomosis. ALT, anterolateral thigh; CMC, carpometacarpal.

**Fig. 13:**
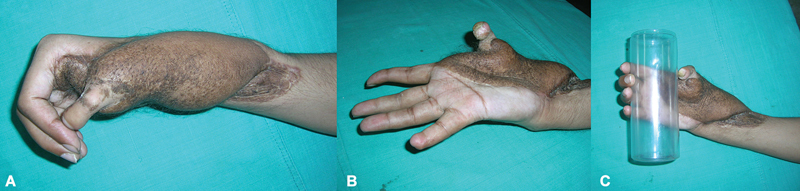
Limited grasp function at follow-up with MPJ instability (same patient as in Fig. 12 ). MPJ, metacarpophalangeal joint.

Further, the possibility of venous congestion is likely to be higher, when this technique is applied to more severe avulsion injuries with skin loss proximal to the distal forearm and even if a successful salvage occurs, it is likely to have a less than optimal return of function. The only patient in the present series with a poor return in sensation and the lowest TAM value had a skin loss extending proximal to the lower third of the forearm. This has also been remarked by Rui et al ( Figs. 12 and 13 ). 5

**Fig. 14:**
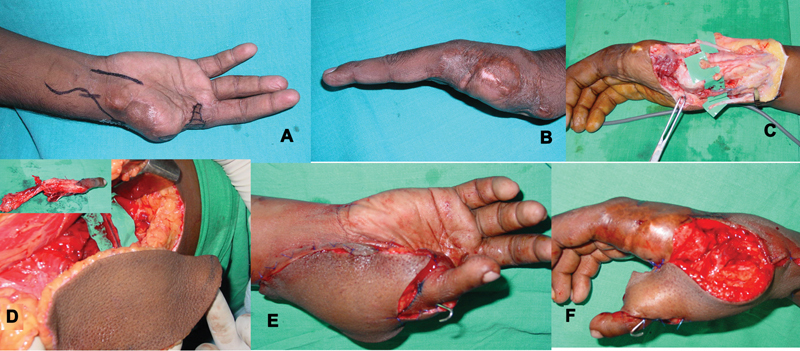
( **A** , **B** ) Preoperative views of thumb loss with absent first web; ( **C** ) recipient area preparation on the radial side of the hand; ( **D** ) ALT flap and second toe harvest; ( **E** , **F** ) dorsal and volar views of the completed reconstruction. ALT, anterolateral thigh.

**Fig. 15:**
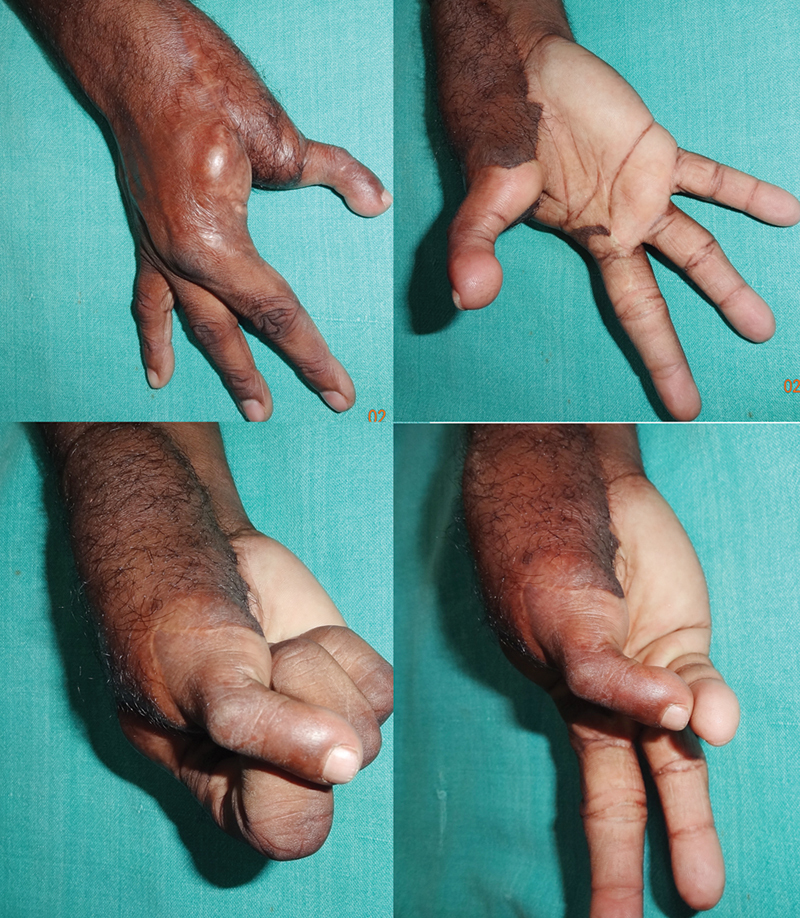
Follow-up images of the patient in Fig. 10 showing span grasp, good thumb rotation, and Kapandji score of 6.

When the thumb is amputated proximal to the MPJ, the ability to rotate the neo-thumb rather than the individual movements at the IPJ and the MPJ is what restores functionality. The less than optimal movements at the IPJ and the MPJ (TAM) has been described in a review article by Lin at al where they note a mean TAM of between 58 and 65 degrees only ( Figs. 14 and 15 ).

They also mention there is no statistical difference in functional results between first and second toe transfers. As regards patient satisfaction, the first toe transfer scores better at the recipient site and the second toe at the donor site. 15

**Fig. 16:**
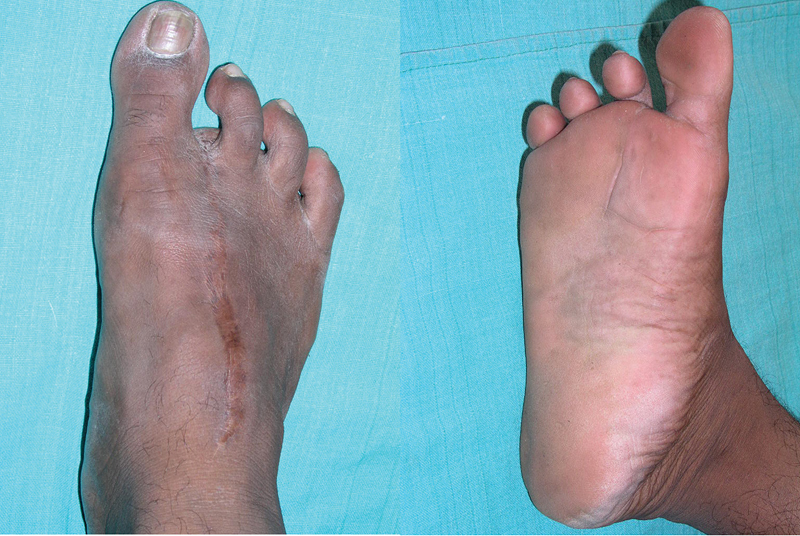
Dorsal and plantar views of foot after second toe transfer at follow-up.

The first toe especially the trimmed toe variant is cosmetically the better for choice for thumb reconstruction but we felt the use of the second toe, with the implication of primary donor-site closure leaving a foot amenable to using “Indian” style footwear, made it easy to convince the patient about the complex procedure ( Fig. 16 ).

Eight of the cases in the present series were amputations through the metacarpal (proximal or middle) and the primary aim of the toe transfer was to restore an opposable digit rather than improve appearance.

Useful points to expedite successful reconstruction:
  - Debridement and fixation at least 12 hours before the major reconstruction.
  - Choosing only patients with injury localized to one upper extremity.
  - Having at least two independent surgical teams during the definitive operation to keep the operative time within safe limits.
  - Choosing the ALT for larger and the extended lateral arm for the smaller defects—keeping the choice of flap to not more than two options will likely increase predictability of success across the series.
  - The trade-off for the second toe rather than the first toe provides less donor morbidity with similar function though the cosmetic appearance is unsatisfactory.
  - Avoiding using tandem flap cover and toe transfer for injuries that have loss of skin proximal to the distal third of the forearm to prevent either flap losses and/or a poor functional result.

## Conclusion

Injuries of the radial side of the hand with skin loss and thumb amputation afford an opportunity to offer an immediate toe transfer. The limitations of recruiting extra skin with the toe safely implies the use of a second flap for skin defect coverage, as a microvascular flap. The toe with the ALT or the extended lateral arm flap affords the possibility of various combinations of piggy-back vascular anastomosis. This telescopes the rehabilitation period and probably makes the surgical procedure easier by avoiding secondary toe transfer in a scarred field.

The second toe donor site being “hidden” is likely to be a more “sellable proposition” to a patient and can end up providing very useful grasp and grip for activities of daily living and thus guarantees he or she is successfully rehabilitated.
